# Increased Collagen Synthesis Rate during Wound Healing in Muscle

**DOI:** 10.1371/journal.pone.0058324

**Published:** 2013-03-19

**Authors:** Shaobo Zhou, Jonathan Salisbury, Victor R. Preedy, Peter W. Emery

**Affiliations:** 1 Diabetes and Nutritional Sciences Division, King's College London, London, United Kingdom; 2 Department of Histopathology, King's College Hospital, London, United Kingdom; Institute of Molecular and Cell Biology, Singapore

## Abstract

Wound healing in muscle involves the deposition of collagen, but it is not known whether this is achieved by changes in the synthesis or the degradation of collagen. We have used a reliable flooding dose method to measure collagen synthesis rate *in vivo* in rat abdominal muscle following a surgical incision. Collagen synthesis rate was increased by 480% and 860% on days 2 and 7 respectively after surgery in the wounded muscle compared with an undamaged area of the same muscle. Collagen content was increased by approximately 100% at both day 2 and day 7. These results demonstrate that collagen deposition during wound healing in muscle is achieved entirely by an increase in the rate of collagen synthesis.

## Introduction

Wound healing involves a series of processes which have traditionally been divided into four phases, haemostasis, inflammation, proliferation and remodelling [1], [2]. The proliferative phase in particular involves deposition of new protein, to close the wound and re-establish tissue integrity and to replace tissue proteins that have been damaged. Relatively little is known about the changes in the rates of protein synthesis and breakdown that result in this net deposition of protein. We have previously measured the rate of protein synthesis in muscle during the healing of a surgical wound *in vivo* and found a substantial increase (70 – 150%), starting 48 hours after the operation and continuing at least until day 7 [3], [4]. Moreover, this increase in protein synthesis was not affected by malnutrition [4], suggesting that increased protein synthesis has a high biological priority. However, it is not known which proteins are involved in this accelerated protein synthesis, nor indeed whether all protein fractions are equally affected.

Much of the new protein deposited during wound healing is collagen, the major protein component of connective tissue. Although collagen was once thought to be subject to minimal turnover, it is now known that collagen synthesis is a dynamic process, with collagen synthesis rates showing considerable variation between different tissues and at different ages [5]–[7]. Moreover, the collagen content of tissues can be controlled by changes in the rates of both synthesis and degradation of collagen [5]–[8].

The aim of the present study was to measure the rate of collagen synthesis in muscle during the healing of a surgical wound at various time points after the surgery. Collagen synthesis rate was measured *in vivo* by injecting a flooding dose of radioactively labelled proline and measuring the increase in specific radioactivity of protein-bound hydroxyproline over the subsequent 30 minutes [8], [9]. Collagen synthesis was also measured simultaneously in undamaged muscle tissue in the same animal, allowing each animal to act as its own control in defining the increase in collagen synthesis rate.

## Materials and Methods

### Ethics statement

All animal procedures were carried out under Home Office Project Licence PPL70/5171 and adhered to institutional guidelines for humane treatment of research animals. Surgery was carried out under general anaesthesia with post-operative analgesia to minimise discomfort and distress to the animals.

### Methods

Twenty four mature female Sprague-Dawley rats (Harlan, Bicester, Oxon, UK) weighing 170–180 g were randomly allocated to three groups. We did not determine or standardise the animals' oestrus cycle. All rats were anaesthetised with isofluorane (3% for induction, 2% for maintenance). A 5 cm midline incision was made through the skin over the abdomen, the skin was freed from the abdominal wall by blunt dissection and a full length incision was made through the abdominal wall. The muscle layer was then closed with a continuous 4/0 silk suture and the skin was closed with stainless steel clips. Buprenorphine (0.01 mg) was injected subcutaneously immediately after the operation and again 8 hours later. After the operation the rats were returned to their cages to recover and were maintained with food and water *ad libitum*.

Collagen synthesis was measured in the three groups of rats 1, 2 and 7 days after surgery using the method of Laurent [9]. A flooding dose of L-[5-^3^H] proline (75 µCi and 1.4 mmol in 0.5 ml per 100 g body weight) was injected via a lateral tail vein and the rats were killed by decapitation 30 minutes later. Blood was collected from the neck into heparinised tubes, centrifuged and plasma was stored frozen for subsequent analysis. A 1 cm-wide strip of abdominal muscle that included the wound site in the middle was rapidly excised. As a control, a similar sized strip was taken from an undamaged area of the abdominal muscle at least 1 cm away from the wound. Tissue samples were rapidly frozen in liquid nitrogen and stored at −70°C for subsequent analysis.

Tissue samples were subsequently thawed and homogenised in ice-cold water containing protease inhibitors (Roche Products Ltd, Welwyn Garden City, Herts, UK), then proteins were precipitated by the addition of ethanol to a final concentration of 67% (v/v) and left to stand at 4°C overnight. The mixture was centrifuged, the supernatant was removed for analysis of free proline specific radioactivity and the protein pellet was washed twice with 67% (v/v) ethanol. The specific radioactivity of free proline in tissue and plasma samples and the specific radioactivity of hydroxyproline in tissue protein were measured by the method of Laurent *et al.* [10].

The fractional rate of collagen synthesis was calculated from the following formula [9]:
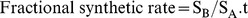
where S_B_ is the specific radioactivity of hydroxyproline in tissue protein, S_A_ is the specific radioactivity of free proline in the tissue and t is the time between injection and killing, nominally 30 minutes but measured separately for each rat.

Hydroxyproline content was measured in two additional groups of ten rats, as an index of collagen content. These rats were treated in exactly the same way as described above and were killed two and seven days after surgery, but without injection of the flooding dose of [^3^H]-proline. Tissue samples were taken from similar areas of abdominal muscle and frozen at −70°C for subsequent analysis. After thawing these samples were homogenised in ice-cold water containing protease inhibitors (Roche Products Ltd, Welwyn Garden City, Herts, UK) and an aliquot was taken for measurement of protein content by the biuret method [11]. The remaining protein was precipitated by addition of 2% (v/v) perchloric acid. After centrifugation, the supernatant was discarded and the pellet was hydrolysed in 6 M HCl for 36 h at 105°C. The acid was removed by placing the tubes under vacuum in a desiccator containing solid NaOH and P_2_O_5_. After drying, the residue was dissolved in sodium citrate (0.5 M, pH 6.3) and aliquots were taken for the measurement of hydroxyproline content by the chloramine T method [12].

Samples from four of the rats that were killed two days after surgery were also taken for histology. Immediately after killing the rats part of the wounded and non-wounded tissue samples was separated and fixed in formalin, then the fixed samples were processed overnight and embedded in paraffin wax according to established procedures [13]. 5 µm sections were then cut on a rotary microtome, stained with haemotoxylin and eosin and coverslipped to allow visualisation under a microscope.

The differences in collagen synthesis rate and content between the wounded and non-wounded areas of the same abdominal muscle were tested using paired t-tests for each of the groups of rats, ie those measured days 1, 2 and 7 days after surgery. Differences between the three groups were tested by one way analysis of variance of the differences in collagen synthesis rate between wounded and non-wounded areas of abdominal muscle, followed by Tukey's post-hoc test. Probabilities less than 0.05 were considered significant. PASW Statistics 18 (SPSS Inc, Chicago, USA) was used for statistical analysis.

## Results

The measured values for the specific radioactivities of both proline and hyroxyproline are shown in **Table 1**. Mean values for the specific radioactivity of free proline in all tissue samples was within 10% of the corresponding value in plasma and also within 10% of the measured value in the injection solution, 74.4 dpm/nmol. There was no significant difference in the specific radioactivity of free proline between the wounded and non-wounded samples or between either tissue and the corresponding plasma value (P>0.05, paired t-tests). This indicates that the tissues were effectively flooded with the precursor amino acid, proline, making the measurements of collagen synthesis rate valid and reliable [8], [9].

**Table 1:** Specific radioactivity of protein-bound hydroxyproline and free proline in abdominal muscle and plasma of rats measured 1, 2 and 7 days after surgery.

	n	Non-wounded muscle	Wounded muscle	Plasma
**Day 1**	5			
Free proline specific radioactivity (dpm/nmol)		73.7 ± 5.5	77.9±6.9	75.3±2.5
Protein-bound hydroxyproline specific radioactivity (dpm/nmol)		0.008±0.001	0.014±0.001	
**Day 2**	6			
Free proline specific radioactivity (dpm/nmol)		79.1±8.6	76.3±2.0	73.0±1.3
Protein-bound hydroxyproline specific radioactivity (dpm/nmol)		0.007±0.002	0.042±0.006	
**Day 7**	8			
Free proline specific radioactivity (dpm/nmol)		68.5±2.7	79.2±2.1	72.3±3.0
Protein-bound hydroxyproline specific radioactivity (dpm/nmol)		0.006±0.001	0.064±0.010	

Values are mean±SE_M_.

**Figure 1:**
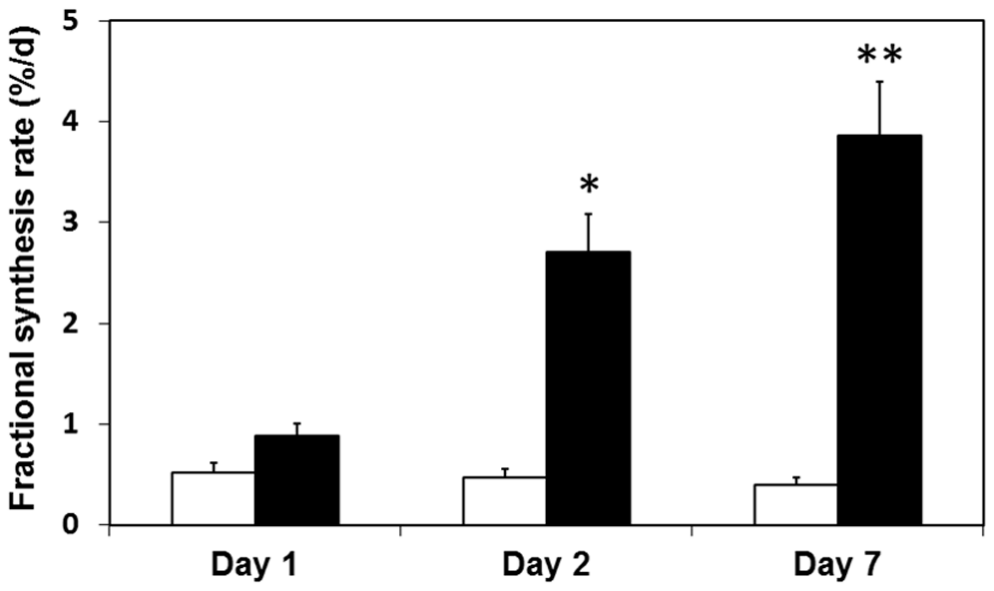
Fractional rates of collagen synthesis in wounded (filled bars) and non-wounded (open bars) areas of abdominal muscle in rats at different times after surgery. Values are means for 5–8 rats per group; error bars represent standard errors of the mean. * Significantly different from corresponding value in non-wounded tissue, P<0.01. ** Significantly different from corresponding value in non-wounded tissue, P<0.001.

The fractional synthesis rates of collagen are shown in **Figure 1**. In comparison with the control (non-wounded) tissue, collagen synthesis in the wounded area of the abdominal muscle showed a non-significant increase on day 1, but was significantly increased by 480% (P = 0.003) on day 2 and 860% (P<0.001) on day 7. One way analysis of variance followed by Tukey's post-hoc test showed that the increase in collagen synthesis rate was greater on days 2 and 7 than that on day 1, but was not significantly different between days 2 and 7.

**Figure 2:**
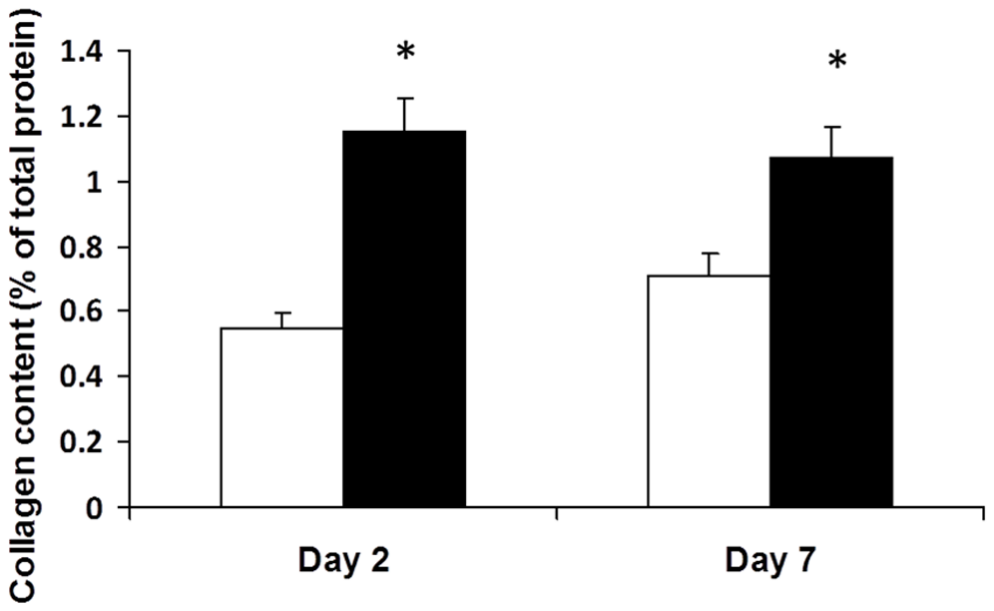
Collagen content of wounded (filled bars) and non-wounded (open bars) areas of abdominal muscle in rats at different times after surgery. Values are means for 10 rats per group; error bars represent standard errors of the mean. * Significantly different from corresponding value in non-wounded tissue, P<0.001.

Results for hydroxyproline content, a measure of the collagen content of the muscle, are shown in **Figure 2** **.** They are expressed as a ratio to protein content in order to normalise for any changes in water content of the wounded muscle. The results show that the collagen content of the wounded muscle was significantly increased by day 2 after wounding and this increase was maintained at day 7.

**Figure 3:**
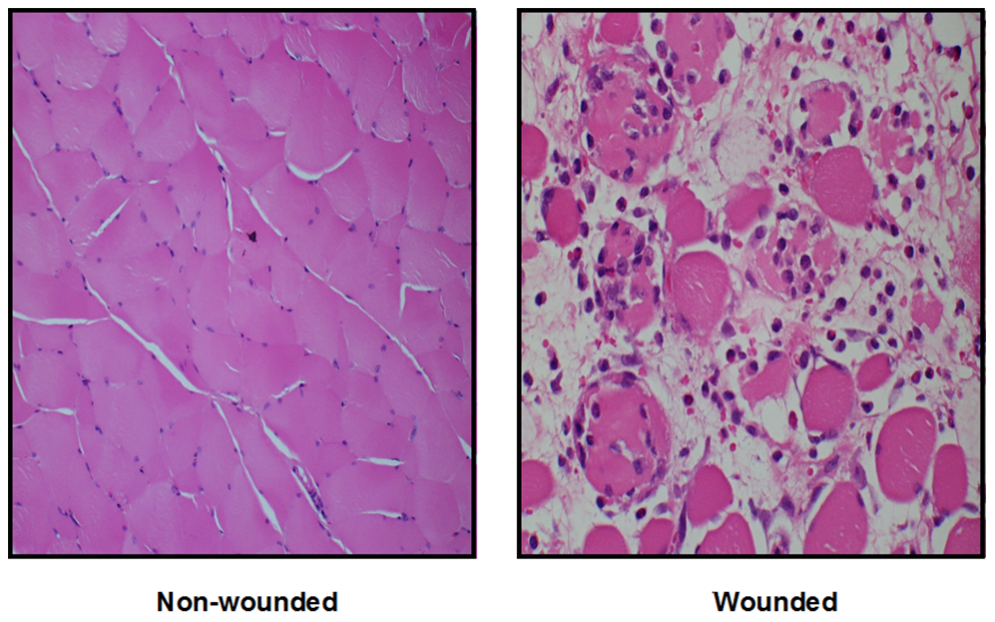
Representative images of wounded and non-wounded tissue two days after surgery. Transverse sections of abdominal muscle stained with haemotoxylin and eosin and visualised at ×200 magnification.

Representative images of the wounded and non-wounded tissues are shown in **Figure 3**, demonstrating that the tissue being analysed was predominantly muscle rather than connective tissue.

## Discussion

We have measured the rate of synthesis of collagen, the most abundant structural protein in muscle, at various stages during the wound healing process. The method used for measuring collagen synthesis rate *in vivo* has been extensively validated in the rat [8], [9]. Its validity depends crucially on all free proline pools in the body being flooded by the large dose of proline that was injected, so that the specific activity of free proline rapidly reaches a steady state value which is similar in all tissues and in the plasma and is maintained relatively constant throughout the 30 minute period of incorporation of labelled proline into protein. This is analogous to the flooding dose phenylalanine method [14] that is commonly used to measure the average synthesis rate of mixed proteins in tissues and indeed was used in our previous experiments to measure muscle protein synthesis rate during wound healing [3], [4]. The effectiveness of the flooding procedure in the present experiment was demonstrated by the equilibration of specific activity of free proline between the muscle samples and the plasma (see **Table 1**).

The results indicate that collagen synthesis rate is considerably increased by the second day after surgery and remains at a high level until at least the seventh day after surgery. These results are broadly in line with our previous data showing an increase in the average rate of synthesis of mixed muscle proteins on days 2 and 7 after surgery [3], [4]. This confirms that collagen synthesis was increased during the proliferative phase of wound healing, which is generally taken as starting on day 2 after wounding [1]. However, the magnitude of the increase in collagen synthesis rate found in the present experiment appeared to be somewhat greater than that of mixed muscle protein synthesis rate found in our previous work (480–860% for collagen *vs* 74–300% for mixed muscle proteins). This raises the question of whether the increased protein synthesis rate that we observed previously could have been accounted for entirely by the increased rate of collagen synthesis. We have not measured the synthesis rates of any other individual protein in this model, although we have begun to develop a method that would allow such measurements to be made [15].

The control of collagen content in tissues is complex, since a high proportion of newly-synthesised collagen appears to be degraded rapidly within the cell [7]. Hence net collagen deposition depends on the balance between collagen synthesis, immediate degradation of newly formed procollagen and longer term degradation of mature collagen by extracellular matrix metalloproteinases [8]. In the present experiment, collagen content was increased by approximately 100% by day 2 after wounding but did not increase further by day 7, while collagen synthesis rate was increased by more than 400% throughout this time. Hence it is likely that the rate of collagen degradation was also increased in response to the large and sustained increase in collagen synthesis.

In summary, this experiment shows for the first time that collagen synthesis in muscle increases on days 2 and 7 after surgery. Our previous work showed that the increase in mixed muscle protein synthesis after surgery was not affected by malnutrition and it would be interesting to see whether the increase in collagen synthesis is equally resistant to the effects of malnutrition.
